# Chondroblastoma: An Unusual Cause Of Shoulder Pain In Adolescence

**DOI:** 10.5334/jbr-btr.1027

**Published:** 2016-02-03

**Authors:** Julie Lambert, Tom Verstraeten, Koen Mermuys

**Affiliations:** 1 University Hospitals Leuven, BE; 2 University Hospitals Ghent, BE; 3 Hospital AZ Sint-Jan AV Bruges, BE

**Keywords:** Chondroblastoma, Shoulder pain, Radiography, Magnetic resonance imaging, Multidetector computed tomography

## Abstract

Chondroblastoma is a rare benign bone tumor, most often localized in the epiphysis of long bones. We report a case of atraumatic shoulder pain in a 17-year old soccer player. This chondroblastoma case demonstrates the difficult differentiation of chondroblastoma from giant cell tumor and clear cell chondrosarcoma and highlights possible pitfalls and clinical importance.

A 17-year-old athletic male presented to the orthopedic center for consultation regarding left shoulder pain lasting more than two months. There was no recent trauma. Clinical examination was unremarkable, with a normal mobility of the shoulder joint. Ultrasound examination of the shoulder was normal. There was no fever or any other sign of systemic illness and laboratory findings were normal. A single corticoid injection was given, which did not result in any pain relief. Subsequently, magnetic resonance (MR) arthrography, performed for the detection of ligamentous or labral injury, revealed a bone lesion located at the epiphysis of the humeral head (Figure 1). The location and morphology of this lesion, as well as the age of the patient, were suggestive for chondroblastoma. However, the hyper-intense signal intensity on the T2-weighted images was a typical for this diagnosis. Additionally, there was only limited surrounding bone marrow edema and no signs of soft tissue inflammation. Plain radiographs were taken immediately following the MR and did show the typical imaging characteristics of a chondroblastoma: a lytic rounded lesion with sharp sclerotic margin (Figure 2).

**Figure 1:**
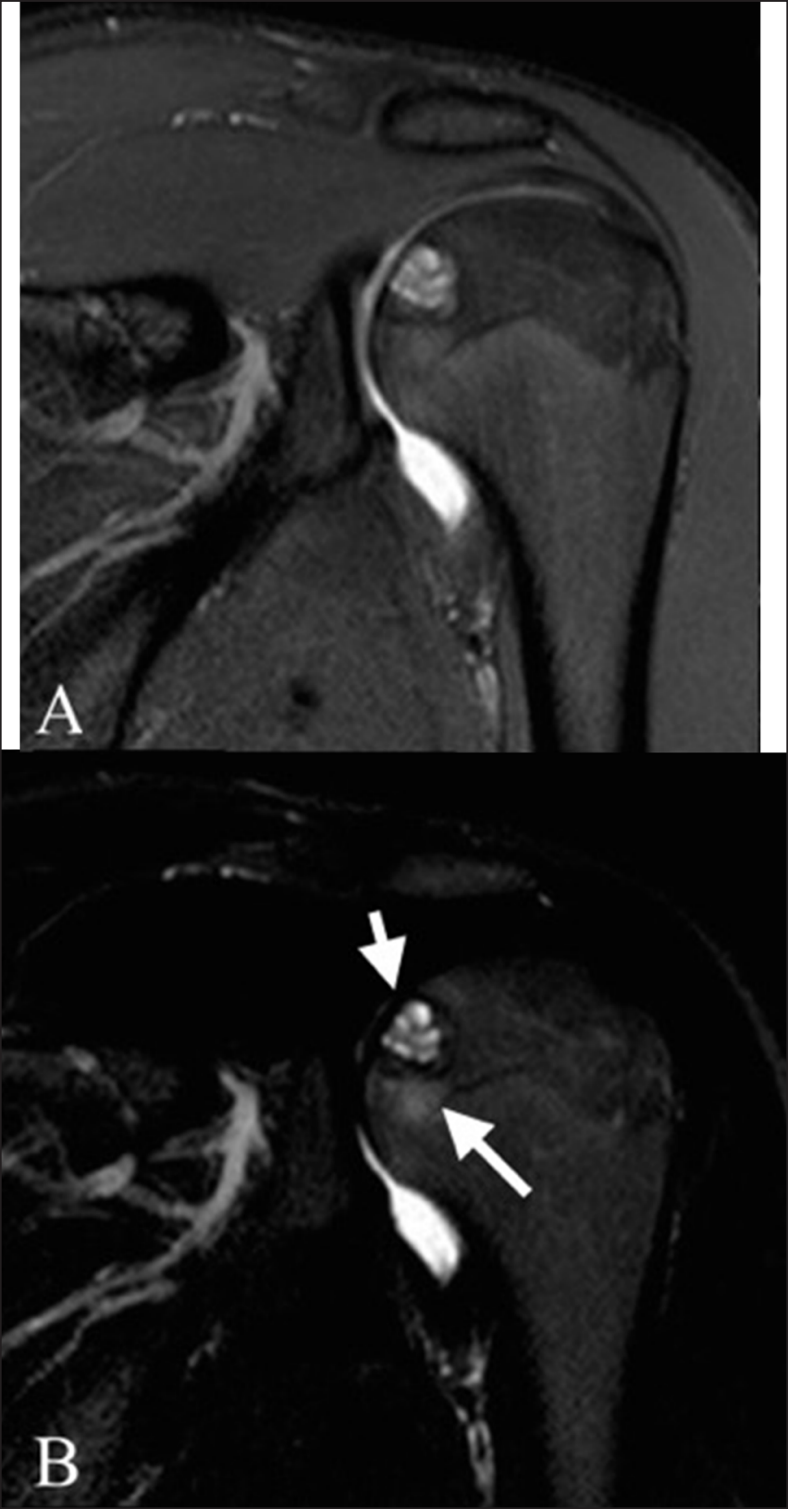
Coronal dual TSE FS images of the left shoulder. (A): proton density weighted image: well described lesion in the proximal humeral epiphysis without disruption of the subchondral bone plate, (B): T2-weighted: central high signal intensity (short arrow), limited bone marrow edema (long arrow).

**Figure 2:**
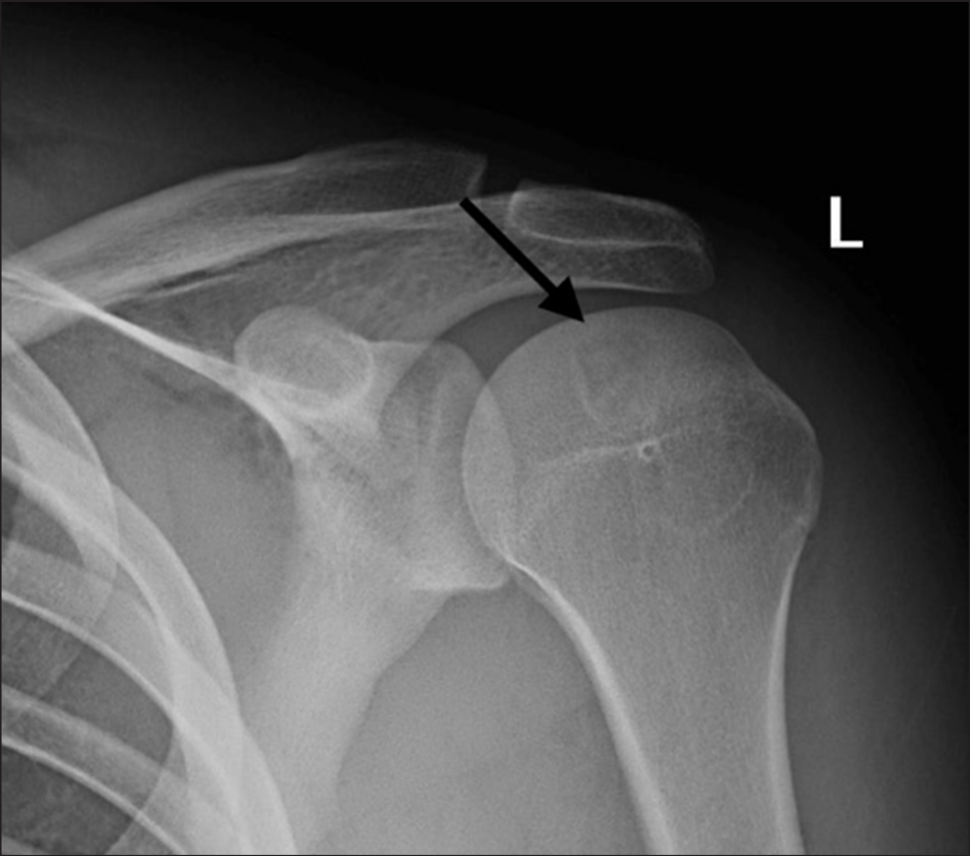
Radiograph of the left shoulder, AP view: a rounded lytic lesion is seen in the epiphysis of the humeral head, with sharp borders and a thin sclerotic rim (arrow).

Central calcifications were suspected on plain films, but much better depicted on computed tomography (Figure 3). Bone scintigraphy did not show increased tracer uptake in the lesion. Curettage was performed and histopathological investigation confirmed the diagnosis of chondroblastoma (Figure 4).

**Figure 3:**
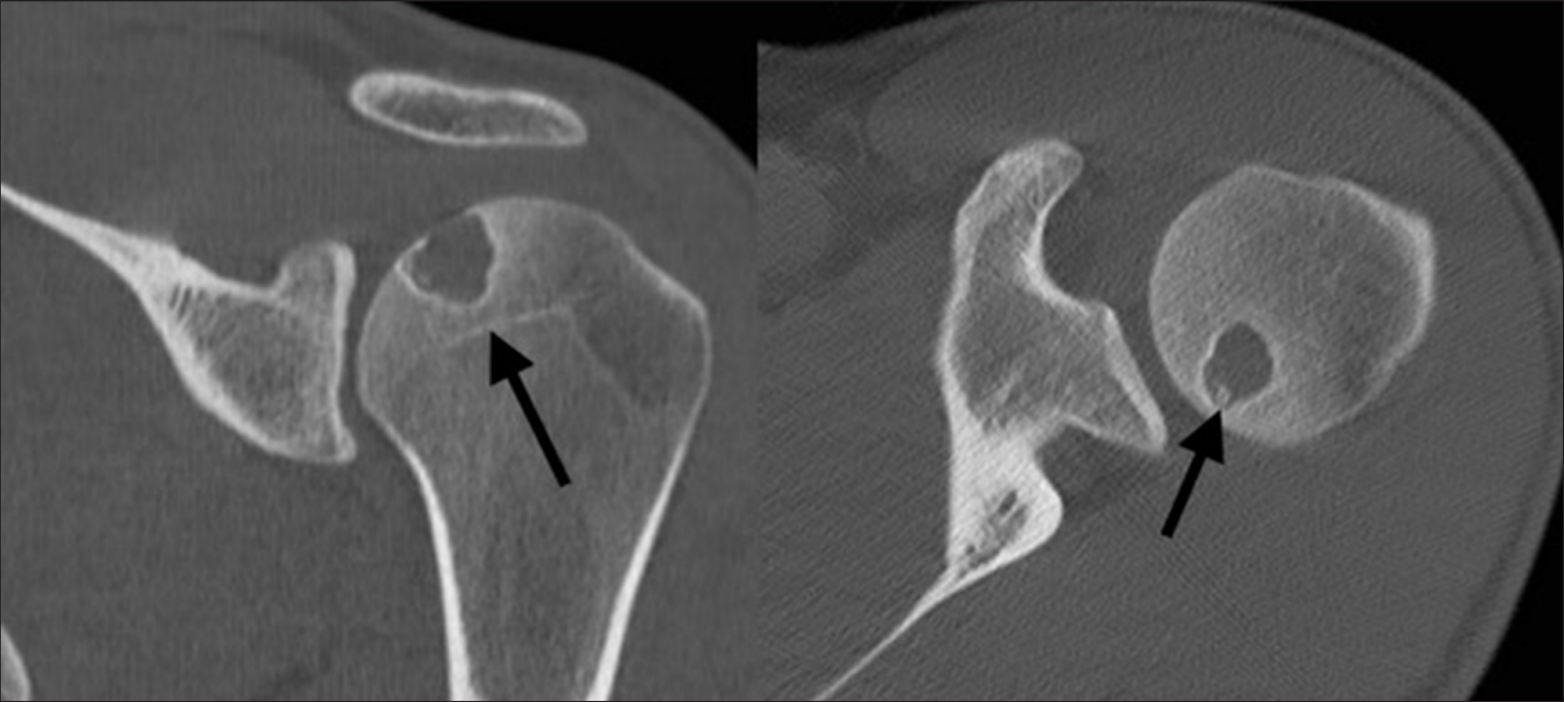
CT of the left shoulder. (A) Coronal reformatted image: the lesion does not reach the growth plate, which is already closed (arrow); (B) Axial plane image: a few small dense fragments can be seen in the lytic lesion (arrow): calcifications, confirming the presence of a chondroid matrix.

**Figure 4:**
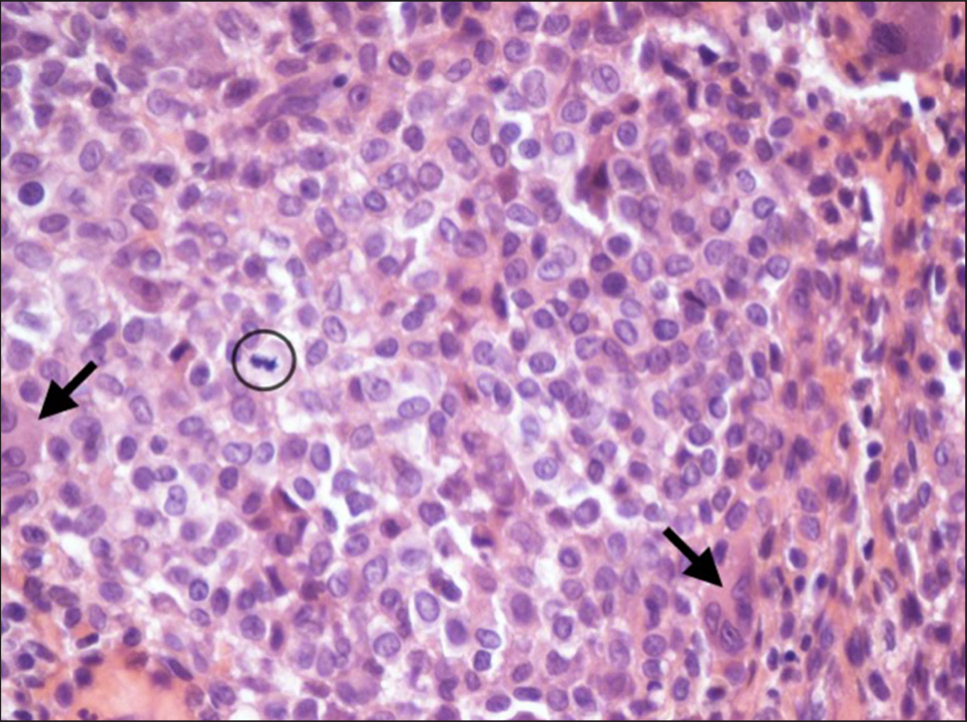
Histological image (HE staining, enlargement 40X) of the resected chondroblastoma: compact rounded tumor cells with bean-shaped nuclei, giant cells (arrows), a mitotic figure (circle) and chondroid matrix (red).

## Discussion

Chondroblastoma is a bone tumor with a prevalence of only 1 to 2% of primary bone tumours [2]. It was first described as a “giant cell tumor with calcifications” by Kolodny in 1927, next as a “calcifying giant cell tumor” by Ewing in 1928 and as an “epiphyseal chondromatous giant cell tumor” by Codman in 1931 [1,2]. It was not until 1942 that it was considered a separate entity by Jaffe and Lichtenstein [1,2]. Onset of pain is usually insidious and patients can also complain about swelling or decreased joint mobility [2]. The only symptom in our patient was pain, which was initially attributed to intensive sports activities. This caused a significant delay to the correct imaging technique.

The first key to the diagnosis is the location. The preferred location of chondroblastoma is the epiphysis of long bones. The differential diagnosis of a tumor or tumor-like lesion located in the epiphysis of a long bone includes: giant cell tumor, clear cell chondrosarcoma, osteomyelitis, intraosseous geode, Paget disease and osteochondral lesion [3]. The clinical presentation mostly makes it possible to exclude several of these diagnoses from the start. The extent of the lesion is a second very important key to the diagnosis. Giant cell tumors, for example, arise in the epiphysis but often grow beyond the growth plate to the metaphysis and are often less well delineated. Chondroblastoma on the other hand, is typically confined to the epiphysis. The third key to diagnosis of chondroblastoma is recognizing the rounded shape, sharp borders and typical sclerotic rim of the lesion on plain radiographs [5]. Computed tomography (CT) can be useful for accurately delineating the borders of the tumor and the distance to the growth plate. Additionally, it allows better visualization of calcifications in the center of the tumor, indicating the chondroid matrix [2,5]. However, this small additional information from CT does not outweigh the much higher radiation dose. Therefore, unlike conventional radiographs, it is not recommended in the diagnostic workup [2].

The bone maturation stage and subsequently the patient age is the fourth important diagnostic clue [2,3]. The tumor is most frequently found in the age group before the second decade, prior to the closure of the growth plates [2,3]. The context of mature skeletal bone usually favors the diagnosis of giant cell tumor over chondroblastoma [3]. Our case shows, however, that this tendency cannot be regarded as an exclusive rule for differentiation, since the growth plate of the humerus was already completely closed.

Another bone tumor with similar age distribution and imaging appearance to chondroblastoma is clear cell chondrosarcoma, a malignant cartilaginous tumor. Differentiation between these two entities is of major clinical importance because while curettage and filling of the defect with bone graft material is sufficient as a treatment for most cases of chondroblastoma, broader surgical resection is warranted in the case of a clear cell chondrosarcoma [4]. Some MR features allow further differentiation between chondroblastoma and chondrosarcoma. First, there is the typical intermediate to low-intensity appearance of chondroblastoma on a T2-weighted sequence as opposed to the characteristic high T2-signal of a clear cell chondrosarcoma. Secondly, chondroblastoma is often surrounded by profound inflammatory changes including bone marrow edema, periosteal reaction and soft tissue changes. However, both these imaging characteristics are not specific and therefore not sufficiently reliable for differentiation between the two tumor types [4]. This is nicely demonstratedin our case, where there was high T2-signal in the lesion and almost no surrounding inflammation. While chondroblastoma is a benign tumor, it can behave very locally aggressive and recurrence after surgery is not uncommon (10–36%) [6]. There is evidence in recent literature that combining the curettage and bone grafting with cryosurgery decreases the recurrence rate of chondroblastoma, although aggressive curettage and bone grafting results in local control in most patients [6].

## Competing Interests

The authors declare that they have no competing interests.
